# Loneliness, subjective health complaints, and medicine use among Finnish adolescents 2006–2018

**DOI:** 10.1177/14034948221117970

**Published:** 2022-08-25

**Authors:** Nelli Lyyra, Niina Junttila, Jorma Tynjälä, Jari Villberg, Raili Välimaa

**Affiliations:** 1 Faculty of Sport and Health Sciences, University of Jyväskylä, Jyväskylä, Finland; 2 Department of Teacher Education, University of Turku, Turku, Finland

**Keywords:** Loneliness, health complaint, medicine use, adolescence, time trend

## Abstract

**Aims:**

Loneliness is an important public health challenge for all ages. This study reports time trends of loneliness among adolescents over a 12-year period and analyses the strength of the associations between loneliness, health complaints, and medicine use.

**Methods:**

Data were derived from the cross-sectional Finnish Health Behaviour in School-aged Children study conducted in 2006, 2010, 2014, and 2018. The study population is based on a random sample of schools with 20,444 participants aged 11–15 years. The trends were analysed with a Mantel–Haenszel test, and the strength of the associations was evaluated by mixed-effects logistic and linear regressions.

**Results:**

An increasing prevalence in frequent loneliness (2006: 11%; 2018: 15%) was evident over the 12-year study period, especially in girls and 15-year-olds. Among all adolescents, loneliness was associated with a higher risk of recurrent health complaints and medicine use to treat the corresponding health issues, especially nervousness (odds ratio 5.8) and sleeping difficulties (odds ratio 7.6).

**Conclusions:**

**Adolescence is a period of higher risk of frequent loneliness and associated health complaints. In this study, loneliness was common among adolescence and an increasing trend of loneliness was observed between 2006 and 2018. Also, psychosomatic health complaints and medicine use were strongly associated with loneliness. Persistent loneliness is a significant health risk and failure to resolve loneliness before entering adulthood may imply significant concerns for future well-being.**

## Background

Satisfying social relationships in adolescence are vital for good mental and physical health. From a public health point of view, adolescence is a crucial period for reducing loneliness and preventing negative health outcomes and social exclusion [1]. Loneliness differs from being alone and is always a negative emotional response to perceived social isolation and longing for human contact [2]. Loneliness is a complicated and multidimensional phenomenon; one close friend may be enough for one individual to avoid feelings of loneliness, whereas a different person may feel lonely despite interacting with numerous friends and a wide social network [3]. Feelings of loneliness are common among school-aged children, especially during adolescence, when developmental changes in companionship, individualisation, identity exploration, and cognitive and physical maturation increase the risk of perceived social isolation and feelings of loneliness [4]. Previous studies have indicated that frequent loneliness becomes more prevalent during adolescence and is typically experienced for a prolonged period [5,6]. In a cross-national comparison study, about 18% of adolescents reported being lonely most or all of the time [7].

Loneliness is associated with various physical and psychological health consequences, especially when it is experienced often or over a prolonged period [8]. Among adolescents, loneliness is associated with a higher prevalence of psychosomatic symptoms [9], increased blood pressure, anxiety, and depression [5]. There is also evidence that loneliness may have long-term health effects; in one study, 11-year-olds who had experienced loneliness at the age of eight had stronger depressive symptoms and poorer general health, took longer to fall asleep, and displayed a higher prevalence of sleep disturbance [10].

Adolescence is also a period in which subjective health complaints become more numerous. Subjective health complaints, which refer to symptoms experienced by an individual with or without a defined diagnosis, reflect emotional distress, behavioural difficulties, and non-specific pain, such as headaches, irritability, and nervousness [11]. A recent meta-analysis of temporal trends in adolescents’ self-reported psychosomatic symptoms from 1980 to 2016 has revealed a minor increase in such symptoms in European countries [12]. Additionally, older adolescents and girls have reported higher levels of recurrent health complaints [13].

In relation to subjective health complaints, self-reported medicine use by adolescents to treat headaches, stomach aches, nervousness, and sleeping difficulties is common across countries [14]. Girls have reported consistently higher frequencies of medicine use for headaches and stomach aches compared to boys, but no consistent gender-related difference is evident in the use of medicine to alleviate nervousness and sleeping difficulties [14,15].

Despite the wide range of research on loneliness, there has been insufficient attention given to the time trends and interactions between loneliness, individual health complaints, and medicine use. There is also a lack of knowledge concerning the extent to which loneliness increases the risk of recurrent individual health complaints and corresponding medicine use. To address these research gaps, this study aims to describe the prevalence and trends of loneliness among 11-, 13-, and 15-year-olds in Finland from 2006 to 2018, and analyse the strength of the associations between loneliness and health complaints, and loneliness and medicine use.

## Methods

### Data collection procedure

Data were collected from Finnish adolescents in 2006, 2010, 2014, and 2018 as part of the Health Behaviour in School-aged Children (HBSC) study. The HBSC study is an international World Health Organization collaborative study that uses cross-sectional surveys performed every four years among students aged 11, 13, and 15. Samples were chosen from the Finnish school register by means of a random cluster sampling method. The sampling was adjusted to take into account the province, municipality, and size of the school. The primary sampling unit was the school. Within the school, the class was randomly selected. The HBSC study protocol ensures that the sample is nationally representative of the target population [16]. The school-level response rates for anonymous and voluntary surveys varied from 64% to 94% in the data collection years. The Finnish National Agency for Education has approved of the Finnish national HBSC study since its inception and covered the data collection in 2006, 2010, and 2014. In 2018, the data were collected electronically. The ethical committee of the University of Jyväskylä reviewed the ethical issues and granted approval.

### Participants

The current study is based on the Finnish national HBSC data collected in 2006, 2010, 2014, and 2018 from Finnish-speaking schools. The total number of participants was 20,444. The sample sizes were 5249, 6723, 5925, and 2547 for the survey years 2006, 2010, 2014, and 2018, respectively. The sample was evenly distributed by gender and age (χ^2^(2) = 1.81; *p* = 0.41); thus, boys and girls comprised similar proportions of each age group. Most participants were European with a Finnish background. The majority were born in Finland, and most were living in urban areas.

### Background variables

Gender, age, data collection year, country of birth, and place of residence were used as background variables to describe the study participants.

### Study variables

Loneliness was measured by a single question on perceived global loneliness. According to previous research, the use of one item to measure loneliness can yield comparable findings to that of the widely used University of California, Los Angeles (UCLA)Loneliness Scale when used as a one-dimensional measure of loneliness [17]. Students indicated the frequency at whi ch they felt lonely by answering the question ‘Do you ever feel lonely?’ with one of the following responses: ‘yes, very often’, ‘yes, quite often’, ‘yes, sometimes’, or ‘never’. The options ‘yes, quite often’ and ‘yes, very often’ were both considered indicative of frequent loneliness.

Subjective health complaints were measured with the HBSC symptom checklist (HBSC-SCL), which is a non-clinical measure of perceived symptoms [18]. The scale adopted in this study included four symptoms (headache, stomach ache, feeling nervous, and difficulty falling asleep). Participants reported the frequency at which they experienced each symptom in the past six months by answering the question ‘In the last six months, how often have you had the following?’ with response options ranging from ‘about every day’ to ‘rarely or never’. Based on previous literature in which recurrent health complaints indicated a higher risk of negative health consequences [19], the individual health complaints in this study were dichotomised as either ‘at least once a week’ or ‘less than once a week’. A standardised sum score was calculated.

Medicine use was examined for each ailment in 13- and 15-year-olds (i.e. medicine use for headaches, stomach aches, difficulty falling asleep, and nervousness). The medicine use items employed in this study have been validated by parental reports [20]. Medicine use was measured with the question, ‘During the last month have you taken any medicine or tablets for the following?: headache; stomach ache; difficulty falling asleep; or nervousness’. The response options were ‘no’, ‘yes, once’, and ‘yes, more than once’. The responses were recorded to reflect the presence or absence of medicine use in the last month (yes/no). A standardised sum score for medicine use was calculated.

### Statistical analysis

Descriptive statistics were used to examine the prevalence and time trends of loneliness. Linear time trends were tested by the Mantel–Haenszel test for trends. For all analyses, a conservative *p*-value of less than 0.001 was considered statistically significant to avoid type-I error. Descriptive analyses were conducted with SPSS 26.0 (SPSS Inc., Chicago, IL, USA).

Mixed-effects logistic regression was first used to separately analyse the strength of associations between loneliness and individual health complaints, and loneliness and each medicine use. Then, mixed-effects linear regression was conducted to investigate the relationships between loneliness and the standardised sum score for health complaints and loneliness, and the standardised sum score for medicine use. The effects of gender, age, and data collection year were investigated with the models by including the variables as independent variables in the regression model. Mixed-effects modelling was utilised for the analysis in view of the clustering of students in classes. The sampling procedure was carried out by school level, and only one class participated from each school. Class was set as a random effect in the mixed-effects equations. The odds ratio (OR) with a corresponding 95% confidence interval (CI) and *p*-value was calculated for each association. The goodness-of-fit of the mixed-effects models was tested against that of the fixed-effects models. A significant chibar^2^ test value indicated that the mixed-effects model had a better fit than the fixed-effects model. The mixed-effects logistic regression and mixed-effects linear regression were conducted with Stata 16 (StataCorp LLC, College Station, TX, USA).

## Results

**Figure 1.**
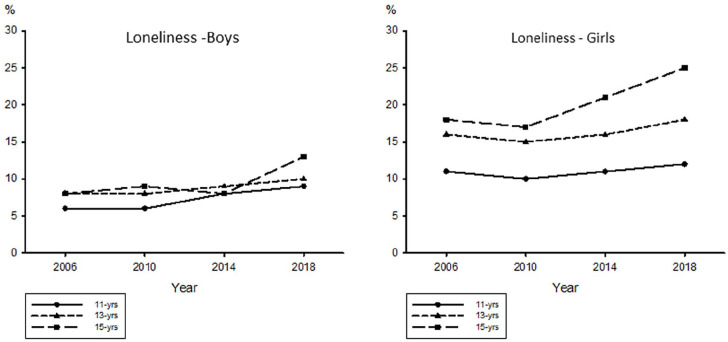
Time trends for 11-, 13-, and 15-year-old male and female adolescents who reported feeling lonely quite often or very often.

Table I displays the prevalence of frequent loneliness in 11-, 13-, and 15-year-old boys and girls at four time points (2006, 2010, 2014, 2018). In the total sample, frequent loneliness increased from 11% to 15% over the 12-year study period. The prevalence of frequent loneliness was higher among girls than boys at each data collection point. It increased from 15% in 2006 to 19% in 2018 among girls and from 7% in 2006 to 10% in 2018 among boys. Frequent loneliness was particularly prevalent among 15-year-olds – especially 15-year-old girls, of whom 25% reported experiencing frequent loneliness in 2018. An increasing linear trend over the 12-year period was observed in the total sample, in 15-year-olds, in boys and girls (all ages combined), and in 15-year-old girls (Table I, Figure 1).

**Table I.** Trends in the prevalence (%) of self-reported loneliness (quite often or very often) and subjective health complaints (once a week or more often) by study year, gender, and age.

Loneliness^c^	Survey year				Difference^a^/*p* for trend^b^
	2006 % [95% CI]	2010 % [95% CI]	2014 % [95% CI]	2018 % [95% CI]	
Boys					
11-year-olds	6.4 [4.7–8.0]	5.9 [4.6–7.3]	7.9 [6.3–9.6]	8.9 [6.2–11.5]	+2.5/*p* = 0.033
13-year-olds	7.5 [5.7–9.3]	7.6 [6.0–9.2]	9.0 [7.2–10.9]	9.4 [6.7–12.1]	+1.9/*p* = 0.131
15-year-olds	8.5 [6.5–10.5]	9.2 [7.4–11.1]	8.5 [6.7–10.3]	13.0 [9.9–16.1]	+4.5/*p* = 0.054
Girls					
11-year-olds	11.1 [9.1–13.1]	10.1 [8.5–11.8]	11.2 [9.3–13.1]	12.8 [9.8–15.9]	+1.7/*p* = 0.343
13-year-olds	15.2 [12.8–17.5]	14.6 [12.5–16.7]	16.0 [13.7–18.4]	17.5 [13.9–21.0]	+2.3/*p* = 0.230
15-year-olds	18.6 [16.1–21.2]	16.5 [14.3–18.7]	20.8 [18.3–23.3]	25.2 [21.3–29.1]	+6.6/***p* = 0.001**
Total					
Boys	7.4 [6.4–8.4]	7.5 [6.6–8.4]	8.5 [7.5–9.5]	10.4 [8.8–12.1]	+3.0/***p* = 0.001**
Girls	14.9 [13.5–16.2]	13.6 [12.5–14.7]	15.9 [14.6–17.2]	18.6 [16.5–20.7]	+3.7/***p* = 0.001**
11-year-olds	8.8 [7.5–10.1]	8.1 [7.0–9.2]	9.6 [8.3–10.9]	10.9 [8.9–12.9]	+2.1/*p* = 0.043
13-year-olds	11.5 [9.9–13.0]	11.3 [9.9–12.6]	12.5 [11.0–14.0]	13.5 [11.3–15.8]	+2.0/*p* = 0.075
15-year-olds	13.9 [12.2–15.6]	13.1 [11.6–14.5]	14.9 [13.3–16.5]	19.4 [16.9–22.0]	+5.5/***p* < 0.001**
**Total sample**	11.3 [10.4–12.2]	10.7 [9.9–11.4]	12.3 [11.4–13.1]	14.7 [13.3–16.0]	+3.4/***p* < 0.001**

Bold indicates significant *p*-values for linear trends.

a Difference between 2006 and 2018 in prevalence of frequent loneliness.

b *p*-value for linear-by-linear association.

c Frequent loneliness: very often and quite often reported feelings of loneliness.

The strength of the associations between loneliness and health complaints and loneliness and medicine use were analysed by mixed-effects logistic regression and mixed-effects linear regression. Frequent loneliness was associated with a higher risk of experiencing health complaints at least once a week as well as the use of medicine to treat the corresponding issues. For students who reported being lonely very often, OR values were significantly higher for headache (3.0), stomach ache (3.8), nervousness (7.6), and sleeping difficulties (5.8) compared to adolescents who were never lonely (Table II). Furthermore, the risk of experiencing weekly health complaints was higher for girls (OR 1.2–1.6) and adolescents aged 13 and 15 (OR 1.4 and OR 1.5, respectively). The survey year 2018 was also associated with a higher risk of experiencing weekly nervousness (OR 1.2) and sleeping difficulties (OR 1.4) compared to 2006 as the reference year. All explanatory variables included in the model (loneliness, gender, age, survey year) were associated with a higher symptom sum score (Table II).

**Table II.** Multilevel linear regression analysis and logistic regression analysis of associations between explanatory variables and symptoms.

	Mixed-effects logistic regression				Linear multilevel regression
	Headache	Stomach ache	Nervousness	Sleeping difficulties	Symptom sum (stand. Z-score)
	OR [95% CI]	OR [95% CI]	OR [95% CI]	OR [95% CI]	OR [95% CI]^a^
*Loneliness*					
Never	1	1	1	1	1
Sometimes	**1.62 [1.52, 1.73]**	**1.74 [1.60, 1.88]**	**2.31 [2.17, 2.46]**	**2.07 [1.94, 2.22]**	**1.54 [1.50, 1.59]**
Quite often	**2.61 [2.32, 2.92]**	**2.90 [2.56, 3.29]**	**5.97 [5.27, 6.76]**	**4.44 [3.95, 4.99]**	**2.58 [2.45, 2.72]**
Very often	**3.01 [2.59, 3.50]**	**3.78 [3.23, 4.41]**	**7.62 [6.41, 9.06]**	**5.77 [4.94, 6.75]**	**3.55 [3.32, 3.79]**
*Gender*					
Boy	1	1	1	1	1
Girl	**1.62 [1.53, 1.73]**	**1.57 [1.46, 1.69]**	**1.26 [1.18, 1.33]**	**1.20 [1.13, 1.28]**	**1.30 [1.26, 1.33]**
*Age*					
11	1	1	1	1	1
13	**1.44 [1.34, 1.56]**	1.10 [1.01, 1.21]	**1.27 [1.18, 1.37]**	**1.21 [1.12, 1.31]**	**1.20 [1.16, 1.24]**
15	**1.50 [1.39, 1.63]**	1.04 [0.95, 1.14]	**1.24 [1.15, 1.35]**	**1.21 [1.12, 1.31]**	**1.21 [1.17, 1.25]**
*Year*					
2006	1	1	1	1	1
2010	0.95 [0.88, 1.03]	1.04 [0.95, 1.15]	0.96 [0.89, 1.05]	1.05 [0.96, 1.14]	1.00 [0,97, 1.04]
2014	1.08 [0.99, 1.17]	1.05 [0.95, 1.16]	1.09 [1.00, 1.18]	1.14 [1.05, 1.25]	**1.07 [1.03, 1.11]**
2018	1.06 [0.96, 1.18]	1.14 [1.01, 1.29]	**1.21 [1.09, 1.35]**	**1.36 [1.22, 1.51]**	**1.13 [1.08, 1.19]**
School level^b^	0.02 [0.01, 0.05]	0.03 [0.01, 0.07]	**0.03 [0.01, 0.06]**	**0.03 [0.01, 0.06]**	**1.01 [1.00, 1,01]**
Chibar^2^	0.008	0.005	**<0.001**	**<0.001**	**<0.001**

Bold indicates a significant association at level *p* < 0.001.

a Regression coefficient values are exponentiated to calculate the corresponding OR values with 95% CI.

b Random effects parameters (var(_cons)). Chibar^2^ test compares the Likelihood Ration (LR) test versus linear model. A significant result indicates that multilevel modelling is significantly better.

Experiencing loneliness very often was also associated with a higher risk of using medicine in the past month to treat a stomach ache (OR 1.5), nervousness (OR 8.4), or sleeping difficulties (OR 6.6; see Table III). In terms of gender, girls reported higher use of medicine for headaches (OR 1.7) and stomach aches (OR 5.7), but lower medicine use for nervousness (OR 0.5). Fifteen-year-olds had a higher risk of using medicine for a headache (OR 1.2) or stomach ache (1.5) or for multiple health complaints (OR 1.2) compared to 13-year-olds. In addition, the survey year 2018 was associated with a higher risk of using medicine for sleeping difficulties (OR 2.8).

**Table III.** Multilevel linear regression analysis and logistic regression analysis of associations between explanatory variables and medicine use.

	Mixed-effects logistic regression				Linear multilevel regression
	Medicine for headache	Medicine for stomach ache	Medicine for nervousness	Medicine for sleeping difficulties	Medicine sum (stand. Z-score)
	OR [95% CI]	OR [95% CI]	OR [95% CI]	OR [95% CI]	OR [95% CI]^a^
*Loneliness*					
Never	1	1	1	1	1
Sometimes	**1.21 [1.12, 1.31]**	**1.25 [1.14, 1.38]**	1.18 [0.87, 1.62]	**1.75 [1.39, 2.20]**	**1.14 [1.10, 1.18]**
Quite often	**1.49 [1.29, 1.72]**	**1.51 [1.29, 1.76]**	**3.26 [2.21, 4.83]**	**3.64 [2.71, 4.89]**	**1.38 [1.30, 1.47]**
Very often	1.11 [0.93, 1.33]	**1.51 [1.24, 1.84]**	**8.43 [5.86, 12.13]**	**6.55 [4.84, 8.85]**	**1.52 [1.40, 1.65]**
*Gender*					
Boy	1	1	1	1	1
Girl	**1.65 [1.53, 1.78]**	**5.65 [5.11, 6.25]**	**0.52 [10.40, 0.68]**	1.03 [0.86, 1.24]	**1.53 [1.48, 1.59]**
*Age*					
11	Na	Na	Na	Na	Na
13	**1**	1	1	1	**1**
15	**1.17 [1.08, 1.26]**	**1.45 [1.335, 1.59]**	1.18 [0.92, 1.52]	1.31 [1.09, 1.58]	**1.18 [1.14, 1.22]**
*Year*					
2006	1	1	1	1	1
2010	1.12 [1.01, 1.24]	**1.18 [1.04, 1.15]**	0.90 [0.63, 1.28]	0.95 [0.71, 1.28]	1.02 [0,97, 1.07]
2014	**1.21 [1.09, 1.34]**	**1.31 [1.16, 1.48]**	0.99 [0.70, 1.40]	**1.82 [1.39, 2.38]**	**1.14 [1.09, 1.20]**
2018	1.05 [0.92, 1.20]	**1.35 [1.16, 1.57]**	1.41 [0.95, 2.09]	**2.79 [2.07, 3.75]**	**1.17 [1.10, 1.24]**
School level^b^	0.04 [0.02, 0.08]	0.03 [0.01, 0.10]	0.14 [0.03, 0.78]	0.12 [0.04, 0.39]	1.00 [1.00, 1,01]
Chibar^2^, *p*	0.001	0.019	0.108	0.030	0.048

Bold indicates a significant association at level *p* < 0.001.

a Regression coefficient values are exponentiated to calculate the corresponding OR values with 95% CI.

b Random-effects parameters (var(_cons). Chibar^2^ test compares the LR test versus linear model.

## Discussion

An increasing linear trend of adolescent loneliness was observed over the 12-year study period. The prevalence of frequent loneliness rose from 11% (2006) to 15% (2018) in the total sample. A linear increasing trend in loneliness was observed in 15-year-olds but not in 13- or 11-year-olds. Loneliness was particularly high among 15-year-old girls, of whom 25% experienced loneliness quite often or very often in 2018. Regression analysis confirmed a strong association between loneliness and subjective health complaints. The results of this study evidence especially strong associations of loneliness with psychological health complaints (nervousness: OR 7.6; sleeping difficulties: OR 5.8) and medicine use targeting those health complaints (nervousness: OR 8.4; sleeping difficulties: OR 6.6).

The finding of an increasing trend in loneliness among 15-year-olds is consistent with recent worldwide Program for International Student Assessment(PISA) survey data. Twenge et al. [21] have noted an increase in adolescent loneliness between 2012 and 2018 in 36 out of 37 countries. Furthermore, the observation of the linear increasing trend among 15-year-olds only (and not 11- or 13-year-olds) complements the previous finding of a stable trend of loneliness among Finnish eight-year-old children over the past 20 years [22]. Potential explanations for the rising trend of loneliness in older adolescents include broader access to smartphones and heavier internet use [21,23]. It is difficult to establish conclusions about the impact of increased internet use on loneliness, as contradictory findings and cross-sectional studies dominate the literature on this topic. Nowland et al. [24] have proposed a bidirectional relationship between loneliness and internet use, whereby the internet can reduce loneliness when used to enhance existing relationships and form new social connections, but can intensify loneliness when used as an escape from the social world. Other research has suggested that the effect of social media use on well-being differs between individuals [25]. Nevertheless, these studies encourage further reflection on the reasons for the different trends in loneliness among children and adolescents and, more specifically, why smartphone access and internet use seem to have a particularly strong impact on older adolescents.

The results of this study also reveal gender-related differences in self-reported loneliness and its associations with subjective health complaints and medicine use. One in four 15-year-old girls reported frequent loneliness, and the regression analysis results highlight that girls generally reported more health complaints and medicine use. These findings correspond with those of cross-national studies on time trends in subjective health complaints, wherein girls and older adolescents displayed higher rates of both individual [14] and multiple health complaints [11]. Moreover, they are in line with a nationwide time trend study in Finland between 2000 and 2011, which showed that a rise in the percentage of girls in adolescent wards was associated with an increase in psychiatric diagnoses [26].

An increasing trend of loneliness was observed among boys as well, although their overall level of loneliness was lower than that of girls. Previous research has suggested that gender-related differences in loneliness could be explained by the fact that indirect scales measuring loneliness tend to indicate higher rates for males, while females are more likely to admit to being lonely when using direct measures and self-labelling [27]. Boys also reported using medicine for nervousness at a higher rate than girls even though the rate of self-reported nervousness was higher among girls. This result might be due to the broad concept of nervousness and variation between individual experiences. In addition, boys in Finland are diagnosed and prescribed medication for attention deficit hyperactivity disorder more often compared to girls [28], which might explain the higher rate at which boys reported using medicine for nervousness. Medicine use is the most common behavioural response to health problems among adolescents, which can be problematic since inappropriate medicine use has been associated with various risk behaviours [29,30]. However, when medication is used under the supervision of a treatment specialist, it can reduce the risk of asocial behaviour or daytime sleepiness.

This study also complements current research on adolescent sleep problems in which adolescents who experienced loneliness very often had a six-fold risk of having sleeping difficulties and a seven-fold risk of taking medication to treat sleeping difficulties compared to adolescents who were never lonely [31]. Sleep disturbance among adolescents can significantly hinder well-being [32], and certain underlying factors, such as smartphone use and social media engagement, might also contribute to more intense feelings of loneliness [33].

Loneliness was associated with a higher risk of all subjective health complaints and medicine use, which can both be problematic. Recurrent somatic symptoms in adolescence can compromise health and negatively affect aspects of everyday functioning, such as school attendance, sleep quality, and the ability to pursue hobbies [34]. In addition, recurrent health complaints during adolescence, such as headaches and sleeping difficulties, may continue into adulthood and develop into more serious health problems [35]. However, the association is bidirectional; health complaints may negatively influence one’s ability to pursue hobbies and attend school, which could feasibly compound loneliness.

The cross-sectional design of this study implies some limitations. This research is based on self-reported survey data collected in 2006, 2010, 2014, and 2018. Since the data are cross-sectional, it is not possible to analyse causal relationships between loneliness, health complaints, and the use of medicine. However, earlier research has indicated that adolescents can reliably report perceived health complaints [18], medicine use [20], and loneliness [36]. This study measured loneliness, subjective health complaints, and medicine use but did not assess underlying or chronic medical conditions that could impact these indicators. The prevalence of chronic conditions in this age group is roughly 15%, which is very close to the prevalence of loneliness reported here. Further research should consider the chronic conditions of adolescents in the analysis to gain a better understanding of medical conditions that may influence the self-reported information.

Moreover, the HBSC survey does not ask respondents if the medicine they used was over-the-counter or prescription. Over-the-counter pain medication is readily available, including for adolescents, and the use of melatonin to treat circadian rhythm disorders and insomnia is becoming more common among children and adolescents [37]. Medicine for nervousness is possibly the only prescription medication that was involved in this study. Further research is needed to assess the role of different types of medicine in adolescent health.

This study measured loneliness with one question: ‘Do you ever feel lonely?’. Based on previous research, the use of one item to measure loneliness can yield comparable findings to that of the widely used UCLA Loneliness Scale when used as a one-dimensional measure of loneliness [17]. While loneliness is a subjective experience that can be reliably measured by self-reporting, the participants in this study may have had varying conceptions of loneliness, and the dimensionality of loneliness could not be evaluated.

This 12-year time trend study highlights an increasing trend in loneliness, especially in 15-year-old girls. Among all adolescents, feelings of loneliness were strongly associated with headaches, stomach aches, nervousness, sleeping difficulties, and the use of medicine to alleviate those symptoms. Frequent loneliness was particularly strongly associated with a higher risk of experiencing nervousness and sleeping difficulties as well as the use of medication to treat these health issues. Although some loneliness during adolescence can be expected, persistent loneliness is not normal and should be identified and addressed as an important health risk. Failure to resolve loneliness before entering adulthood may imply significant concerns for future well-being and health throughout one’s lifespan. From a public health point of view, school-based interventions and health education efforts might be effective ways to mitigate this problem, as they cover the entire adolescent population.
